# Effects of exercise modalities on central hemodynamics, arterial stiffness and cardiac function in cardiovascular disease: Systematic review and meta-analysis of randomized controlled trials

**DOI:** 10.1371/journal.pone.0200829

**Published:** 2018-07-23

**Authors:** Yahui Zhang, Lin Qi, Lisheng Xu, Xingguo Sun, Wenyan Liu, Shuran Zhou, Frans van de Vosse, Stephen E. Greenwald

**Affiliations:** 1 Sino-Dutch Biomedical and Information Engineering School, Northeastern University, Shenyang, Liaoning, China; 2 Key Laboratory of Medical Image Computing, Ministry of Education, Northeastern University, Shenyang, Liaoning, China; 3 Key Laboratory of Cardiovascular Disease, Fuwai Hospital, National Center for Cardiovascular Disease, Chinese Academy of Medical Science, Beijing, China; 4 Department of Biomedical Engineering, Eindhoven University of Technology, Eindhoven, The Netherlands; 5 Blizard Institute, Barts & The London School of Medicine &Dentistry, Queen Mary University of London, London, United Kingdom; Universita degli Studi di Roma La Sapienza, ITALY

## Abstract

**Background:**

Exercise is accepted as an important contribution to the rehabilitation of patients with cardiovascular disease (CVD). This study aims to better understand the possible causes for lack of consensus and reviews the effects of three exercise modalities (aerobic, resistance and combined exercise) on central hemodynamics, arterial stiffness and cardiac function for better rehabilitation strategies in CVD.

**Methods:**

The electronic data sources, Cochrane Library, MEDLINE, Web of Science, EBSCO (CINAHL), and ScienceDirect from inception to July 2017 were searched for randomized controlled trials (RCTs) investigating the effect of exercise modalities in adult patients with CVD. The effect size was estimated as mean differences (MD) with 95% confidence intervals (CI). Subgroup analysis and meta-regression were used to study potential moderating factors.

**Results:**

Thirty-eight articles describing RCTs with a total of 2089 patients with CVD were included. The pooling revealed that aerobic exercise [MD(95%CI) = -5.87 (-8.85, -2.88), P = 0.0001] and resistance exercise [MD(95%CI) = -7.62 (-10.69, -4.54), P<0.00001] significantly decreased aortic systolic pressure (ASP). Resistance exercise significantly decreased aortic diastolic pressure [MD(95%CI) = -4(-5.63, -2.37), P<0.00001]. Aerobic exercise significantly decreased augmentation index (AIx) based on 24-week exercise duration and patients aged 50–60 years. Meanwhile, aerobic exercise significantly improved carotid-femoral pulse wave velocity (cf-PWV) [MD(95%CI) = -0.42 (-0.83, -0.01), P = 0.04], cardiac output (CO) [MD(95% CI) = 0.36(0.08, 0.64), P = 0.01] and left ventricular ejection fraction (LVEF) [MD(95%CI) = 3.02 (2.11, 3.93), P<0.00001]. Combined exercise significantly improved cf-PWV [MD(95%CI) = -1.15 (-1.95, -0.36), P = 0.004] and CO [MD(95% CI) = 0.9 (0.39, 1.41), P = 0.0006].

**Conclusions:**

Aerobic and resistance exercise significantly decreased ASP, and long-term aerobic exercise reduced AIx. Meanwhile, aerobic and combined exercise significantly improved central arterial stiffness and cardiac function in patients with CVD. These findings suggest that a well-planned regime could optimize the beneficial effects of exercise and can provide some evidence-based guidance for those involved in cardiovascular rehabilitation of patients with CVD.

## Introduction

Cardiovascular disease (CVD) is the leading cause of death and the main risk factor for world-wide morbidity [1, 2]. According to the World Health Organization global status report on Non-communicable Diseases (NCD) in 2014, 38 million of the world’s 56 million deaths are from NCDs. Figures from the same report suggest that in 2012 an estimated 17.5 million (46%) of these deaths are due to CVD. Of these deaths from CVD, heart attacks are responsible for 7.4 million, and stroke, for 6.7 million [3]. Therefore, low-cost and effective prevention and treatment are urgently needed.

Insufficient physical activity is considered the fourth leading risk factor for global deaths, and in 2010, was responsible for 69.3 million Disability Adjusted Life Years (DALYs) [3, 4]. Regular physical activity is accepted as an important contribution to the prevention and rehabilitation of CVD [5, 6]. Exercise-based cardiac rehabilitation (aerobic endurance training, dynamic resistance training and both in combination) have been used to manage cardiovascular health in individuals with CVD [6].

Central hemodynamics and arterial stiffness have been recognized as strong independent predictors of all-cause mortality of cardiovascular (CV) events [7–9]. These parameters, such as central blood pressure, augmentation index (AIx) and carotid-femoral pulse wave velocity (cf-PWV)) were used to evaluate the exercise-based rehabilitation of patients with CVD [10–12]. Previous meta-analyses have reported the effects of exercise training on arterial stiffness. However, these analyses mainly focused on a range of adult subjects, including patients with CVD, diabetes, obesity and healthy people [13, 14]; or the effect of one type of exercise training (such as aerobic or resistance exercise) on arterial stiffness [10, 15, 16].

In addition, some studies have investigated the effect of two or three types of exercise training on central hemodynamics and arterial stiffness. Croymans et al. found that aortic systolic blood pressure was decreased, while AIx and cf-PWV were not altered in response to high-intensity resistance exercise [17]. Ashor et al. concluded that AIx and PWV were significantly reduced with aerobic exercise, while resistance or combined exercise had no significant effect on these variables [13]. Figueroa found that resistance exercise had no clear cut effects on central blood pressure and wave reflection in obese adults with prehypertension [18]. There was no consensus about the effects of different exercise modalities on the central hemodynamic and central arterial stiffness variables.

Moreover, central hemodynamics and central arterial stiffness are closely related to cardiac function [19, 20]. Arterial stiffness, central hemodynamics and cardiac function contribute to the complex pathophysiological mechanism of CVD. Increased arterial stiffness (as expressed by PWV) leads to early arrival at the heart of reflected waves from peripheral sites, resulting in augmentation of central systolic pressure. This augmentation of central systolic pressure can lead to adverse changes in cardiac function such as elevation of left ventricular afterload and decreased coronary perfusion [21–23], which may in turn, lead to left ventricular hypertrophy and myocardial ischemia [23, 24]. Previous studies have investigated these changes and reported the effects of exercise training on arterial stiffness/central hemodynamics, cardiac output (CO) and left ventricular ejection fraction (LVEF) in patients with CVD. However, it was controversial for the effects of exercise training on these parameters in CVD. Kitzman et al. found that exercise training did not increase the ejection duration (EF) or improve arterial stiffness [25], and Chrysohoou et al. showed that PWV and LVEF was not improved in response to combined exercise [26]. On the other hand, Molmen-Hansen et al. found that aerobic exercise increased CO, LVEF, and decreased blood pressure and total peripheral resistance (TPR) in patients with hypertension [27]. Understanding of the effects of different exercise training on the central hemodynamics, central arterial stiffness and cardiac function merits comprehensive analysis and evaluation.

Therefore, this systematic review and meta-analysis aimed to investigate the effect of different exercise modalities on central hemodynamics, central arterial stiffness and cardiac function in patients with CVD. Additionally, this meta-analysis provided an overall assessment of effect of different exercise modalities on cardiovascular system to evaluate the possible causes for aforementioned lack of consensus.

## Methods

### Protocol and registration

This meta-analysis was performed according to the guidelines of Preferred Reporting Items for Systematic Reviews and Meta-Analyses (PRISMA). A completed PRISMA checklist was shown in S1 Text. The protocol of this study has been recorded in http://www.crd.york.ac.uk/PROSPERO. PROSPERO registration number: CRD42016052379.

#### Search strategy

The search for relevant studies was performed via electronic searches of five databases (Cochrane Library, MEDLINE, Web of Science, EBSCO (CINAHL), and ScienceDirect from their inception to July 2017). This meta-analysis was only limited to RCTs. The electronic search strategies for all databases are provided in S2 Text. We also searched for eligible articles in reference citation of reviews and research articles.

#### Inclusion criteria

1. Types of studies: Only published RCTs were covered in this meta-analysis.
2. Types of participants: Patients (aged> = 18 years) with CVD were considered, including those with heart pathology (such as coronary artery disease, heart failure, acute myocardial infarction, etc.), hypertension and cerebrovascular disease (stroke).
3. Types of interventions: Patients (exercise-rehabilitation group) undergoing aerobic exercise, resistance exercise, and combined exercise were considered. Control groups (non-exercise group) included those with a sedentary life style and those having some life-style education. In addition, subjects who had had exercise intervention were included if they had been assigned to a control group and compared to others who had undertaken more strenuous exercise.
4. Types of outcome measures: These included central hemodynamic variables (e.g., aortic systolic pressure (ASP), aortic diastolic pressure (ADP), AIx), central arterial stiffness, as expressed by cf-PWV and cardiac function (CO and LVEF).

#### Selection of studies

The same selection criteria were independently used by two authors to screen the titles, abstracts and full texts of relevant studies. Articles that did not meet the inclusion criteria were removed including reviews, non-RCTs, those investigations with only healthy participants, and patients without CVD, CVD with serious arrhythmia or unstable angina, serious aortic stenosis, serious congestive heart failure or pulmonary hypertension with exercise contraindication, less than aerobic exercise intensity, exercise durations with less than 4 weeks, non-exercise intervention, no control groups and non-central hemodynamic or arterial stiffness or cardiac function variables. Any disagreement was discussed or arbitrated by a third author.

#### Data extraction and management

The following information was extracted: study characteristics (e.g., article, year and country), participant characteristics (e.g., age and sample size of different groups), disease type, intervention description, trial period, outcome measures and exercise duration (period of exercise intervention). The two authors who selected the articles also extracted and managed the information therein. Any disagreement was discussed or arbitrated by a third author.

#### Quality assessment

The PEDro scale [28] was used to assess the risk of bias for inclusion in this meta-analysis. This is a free database of randomized trials, systematic reviews and clinical practice guidelines in physiotherapy. The methodological quality of each article was independently evaluated by the two reviewing authors using a total scale (11-item). The following information was assessed: eligibility criteria, point estimates and variability, between-group comparisons, intention-to-treat analysis, adequate follow-up, blinded assessors, blinded subjects, blinded therapists, baseline comparability, concealed allocation and random allocation. When a disagreement occurred, a third author was consulted.

#### Statistical analysis

This meta-analysis used the Review Manager Software (RevMan 5.3) and stata12.0 to analyze data. The *I*^2^ statistic and the chi-square test were used to assess the heterogeneity of the included articles. There was significant heterogeneity when p>0.1 when using the Cochrane Q statistic in the forest plot. The consistency between studies was evaluated by *I*^2^, and risk (low, moderate and high) of heterogeneity was categorized by *I*^2^<25%, *I*^2^ = 25%-75% and *I*^2^>75%, respectively [29]. The outcome measures of each study were combined by meta-analysis using a fixed effects model or random effects model. Given that all the variables from the included articles were continuous, the standardized mean difference (SMD) or the mean difference (MD) and the 95% confidence interval (CI) were used to analyze the studies. Mean and standard deviation (SD) of the variables measured in the RCTs from before and after exercise were included in the forest plots. If the continuous data were summarized by median and interquartile range (IQR), SD was computed as SD = IQR/1.35 [30]. The SD also could be obtained from the equation: $SD=SE\times \sqrt{N}$, where SE is the standard error, and N is the number of participants. P<0.05 was considered as statistically significant [30]. Sensitivity analysis was performed by removing each inclusion article to assess the quality and consistency of results. Subgroup analyses were conducted to investigate the effect of different exercise modalities (aerobic, resistance and combined exercise) on central hemodynamics, arterial stiffness and cardiac function, respectively. Subgroup analyses were also performed to explore the source of heterogeneity according to age, disease, exercise duration and gender. Meta-regression was used to explore the relationships between study characteristics (such as age, disease, exercise duration and gender) and cardiovascular variables by using Stata software (version 12.0). In addition, funnel plot asymmetry estimation was conducted to evaluate possible publication bias using Egger’s regression test [31].

## Results

### Search results

**Fig 1:**
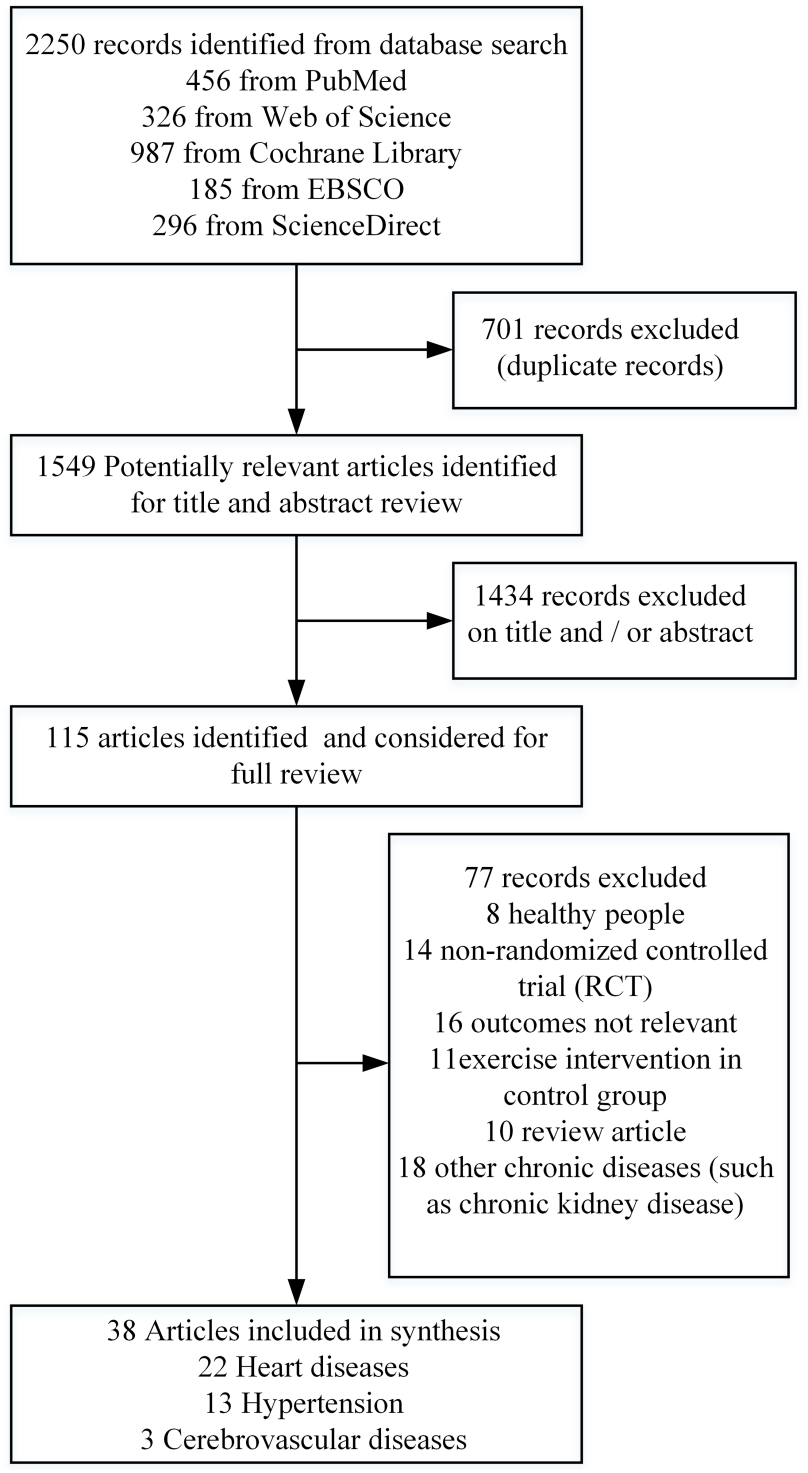
Flow chart of the study selection procedure.

A flow chart describing the selection process is shown in Fig 1. One hundred and fifteen potentially eligible articles were identified from MEDLINE, Web of Science, the Cochrane library, EBSCO and ScienceDirect. After reviewing the full content of these articles, thirty-eight articles satisfied the inclusion criteria. Seventy-seven were excluded for the following reasons: patients free of CVD, non-randomized controlled trials, review articles, other chronic disease (such as chronic kidney disease, type 2 diabetes or rheumatoid arthritis), articles having irrelevant outcomes (such as peripheral circulatory variables), or interventions affecting the control group (such as diet or drug) which made them unsuitable. The basic characteristics of each study are summarized in Table 1. The thirty-eight included articles [11, 25–27, 32–65] covered 2089 patients with CVD (22 articles with heart disease, 13 with hypertension, and 3 with cerebrovascular disease). The distribution of articles by country of publication was: United States (n = 11, 28.95%), the United Kingdom (n = 3, 7.9%), Canada (n = 3, 7.9%), Italy (n = 4, 10.53%), China (n = 3, 7.9%), Brazil (n = 1, 2.63%), Germany (n = 2, 5.26%), South Korea (n = 1, 2.63%), Denmark (n = 1, 2.63%), Turkey (n = 2, 5.26%), Australia (n = 1, 2.63%), Portugal (n = 1, 2.63%), Greece (n = 1, 2.63%), Norway (n = 1, 2.63%), Belgium (n = 1, 2.63%), Poland (n = 1, 1. 2.63%), and Switzerland (n = 1, 2.63%).

**Table 1:** Characteristics of the included studies.

Article, year	Country	Participant Characteristic, Sample Size	Disease	Intervention/comparison groups	Duration of trial period	Outcomes	Time point
**Aerobic exercise**							
Acar (2015) [33]	Turkey	54 subjects (G1 = 27, G2 = 27). Mean age: G1 = 57 years, G2 = 57 years	Acute myocardial infarction	G1: Cardiac rehabilitation exercise program G2: No intervention	Three to four times weekly for 3 months	LVEF	3 months
Aksoy (2015) [35]	Turkey	38 subjects (G1 = 19, G2 = 19). Mean age(SD): G1 = 63.7 years (8.8), G2 = 57.5 years (11.2)	Heart failure	G1: Intermittent aerobic exercise G2: No intervention	Three times a week for 10 weeks	LVEF	10 weeks
Aksoy (2015) [35]	Turkey	38 subjects (G1 = 19, G2 = 19). Mean age(SD): G1 = 59.6 years (6.9), G2 = 57.5 years (11.2)	Heart failure	G1: Moderate-intensity continuous aerobic exercise G2: No intervention	Three times a week for 10 weeks	LVEF	10 weeks
Andersen (2014) [36]	UK	33 subjects (G1 = 21, G2 = 22). Mean age(SD): G1 = 45.8 years (7.2), G2 = 46.9 years (7.6)	Hypertensive men	G1: Football exercise G2: No intervention	Two times per week for 3 months	LVEF	3 months
Andersen (2014) [36]	UK	33 subjects (G1 = 21, G2 = 22). Mean age(SD): G1 = 45.8 years (7.2), G2 = 46.9 years (7.6)	Hypertensive men	G1: Football exercise G2: No intervention	Two times per week for 6 months	LVEF	6 months
Beck (2013) [37]	USA	28 subjects (G1 = 13, G2 = 15). Mean age(SD): G1 = 20.1 years (3.2), G2 = 21.6 years (3.1)	Prehypertensive subjects	G1: Endurance exercise G2: No intervention	Three days per week for 8 weeks	ASP, ADP, AIx, cf-PWV	8 weeks
Beer (2008) [38]	UK	24 subjects (G1 = 12, G2 = 12). Mean age(SD): G1 = 52.8 years (12), G2 = 58.2 years (16)	Patients with dilated cardiomyopathy	G1: Exercise training G2: No intervention	Five sessions/week for 2 months	LVEF	2 months
Beer (2008) [38]	UK	24 subjects (G1 = 12, G2 = 12). Mean age(SD): G1 = 52.8 years (12), G2 = 58.2 years (16)	Patients with dilated cardiomyopathy	G1: Exercise training G2: No intervention	Five sessions/week for 8 months	LVEF	8 months
Belardinelli (1995) [39]	USA	27 subjects (G1 = 18, G2 = 9). Mean age(SD): G1 = 56 years (7), G2 = 57 years (6)	Heart failure	G1: Exercise training G2: No intervention	Three times a week for 12 weeks	LVEF	12 weeks
Blumenthal (2000) [41]	USA	78 subjects (G1 = 54, G2 = 24). Mean age(SD): G1 = 46.6 years (1.2), G2 = 47.2 years (1.8)	Mild hypertension	G1: Aerobic training G2: No intervention	Three to four times per week for 6 months	CO	6 months
Brubaker (2009) [42]	USA	59 subjects (G1 = 30, G2 = 29). Mean age(SD): G1 = 70.4 years (5.3), G2 = 69.9 years (6.3)	Heart failure	G1: Aerobic exercise program G2: No intervention	Three times per week for 16 weeks	LVEF	16 weeks
Donley (2014) [43]	USA	22 subjects (G1 = 11, G2 = 11). Mean age :G1 = 46 years, G2 = 44 years	Metabolic syndrome	G1: Aerobic exercise G2: No intervention	Three times a week for 8 weeks	ASP, AIx, cf-PWV	8 weeks
Dubach (1997) [44]	USA	25 subjects (G1 = 12, G2 = 13). Mean age(SD): G1 = 56 years (5), G2 = 55 years (7)	Myocardial infarction	G1: Aerobic exercise G2: Usual clinical follow-up	45 min periods/week for 8 weeks	CO, LVEF	8 weeks
Dubach (1997) [44]	USA	25 subjects (G1 = 12, G2 = 13). Mean age(SD): G1 = 56 years (5), G2 = 55 years (7)	Myocardial infarction	G1: Aerobic exercise G2: Usual clinical follow-up	45 min periods a week for 2 months	CO, LVEF	2 months
Faulkner (2016) [46]	UK	47 subjects (G1 = 25, G2 = 22). Mean age(SD): G1 = 66 years (12), G2 = 68 years (10)	Stroke	G1: Aerobic exercise G2: Usual care	Twice weekly for 12 weeks	AIx, ASP	12 weeks
Fu (2013) [48]	Taiwan, China	30 subjects (G1 = 15, G2 = 15). Mean age(SD): G1 = 67.5 years (6.97), G2 = 67.8 years (9.68)	Heart failure	G1: Aerobic interval training G2: General healthcare	Three days/week for 12 weeks	LVEF, CO	12 weeks
Fu (2013) [48]	Taiwan, China	30 subjects (G1 = 15, G2 = 15). Mean age(SD): G1 = 66.3 years (8.13), G2 = 67.8 years (9.68)	Heart failure	G1: Moderate continuous training G2: General healthcare	Three days/week for 12 weeks	LVEF, CO	12 weeks
Giallauria (2013) [49]	Italy	46 subjects (G1 = 25, G2 = 21). Mean age(SD): G1 = 54 years (7), G2 = 54 years (9)	Acute myocardial infarction	G1: Multi-comprehensive exercise intervention G2: General healthcare	Three times/week for 6 months	LVEF	6 months
Giannuzzi (2003) [50]	Italy	90 subjects (G1 = 45, G2 = 45). Mean age(SD): G1 = 54 years (7), G2 = 54 years (9)	Chronic heart failure	G1: Moderate continuous training G2: Educational support	Three to five times a week for 6 months	LVEF	6 months
Guimaraes (2010) [51]	Brazil	27 subjects (G1 = 16, G2 = 11). Mean age(SD): G1 = 50 years (8), G2 = 47 years (6)	Hypertension	G1: Continuous exercise training G2: Sedentary routine	Two 40-min sessions a week for 16 weeks	cf-PWV	16 weeks
Guimaraes (2010) [51]	Brazil	27 subjects (G1 = 16, G2 = 11). Mean age(SD): G1 = 45 years (9), G2 = 47 years (6)	Hypertension	G1: Interval exercise training G2: Sedentary routine	Two 40-min sessions a week for 16 weeks	cf-PWV	16 weeks
Hambrecht (2000) [52]	Germany	73 subjects (G1 = 36, G2 = 37). Mean age(SD): G1 = 54 years (9), G2 = 55 years (8)	Chronic heart failure	G1: Ergometer exercise training G2: No intervention	Four to six times per day in 2 week for 6 months	CO, LVEF	6 months
Huang (2014) [53]	Taiwan, China	66 subjects (G1 = 33, G2 = 33). Mean age(SD): G1 = 60 years (17.22), G2 = 56 years (22.96)	Heart failure	G1: Modified high-intensity interval training G2: Usual healthcare	Three days/week for 8 weeks	CO	8 weeks
Iellamo (2000) [54]	Italy	86 subjects (G1 = 45, G2 = 41). Mean age(SD): G1 = 59.4 years (7.8), G2 = 58.5 years (7.3)	Coronary artery disease	G1:Cardiac rehabilitation G2: Usual healthcare	2–4 times for 4 weeks	ASP, ADP	4 weeks
Kitzman (2013) [25]	Canada	63 subjects (G1 = 32, G2 = 31). Mean age(SD): G1 = 70 years (7), G2 = 70 years (7)	Heart failure	G1: Endurance exercise training G2: Attention control	Three times per week for 16 weeks	LVEF	16 weeks
Krustrup (2013) [55]	Denmark	33 subjects (G1 = 22, G2 = 11). Mean age :G1 = 46 years, G2 = 46 years	Hypertensive men	G1: Soccer training G2: Doctor advice	Two sessions per week for 3 months	AIx	3 months
Krustrup (2013) [55]	Denmark	33 subjects (G1 = 22, G2 = 11). Mean age :G1 = 46 years, G2 = 46 years	Hypertensive men	G1: Soccer training G2: Doctor advice	Two sessions per week for 6 months	AIx	6 months
Madden (2013) [57]	Canada	52 subjects (G1 = 26, G2 = 26). Mean age(SD): G1 = 68.5 years (4.6), G2 = 70 years (4.1)	Hypertension	G1: Vigorous aerobic exercise G2: No intervention	Three times per week for 3 months	cf-PWV	3 months
Madden (2013) [57]	Canada	52 subjects (G1 = 26, G2 = 26). Mean age(SD): G1 = 68.5 years (4.6), G2 = 70 years (4.1)	Hypertension	G1: Vigorous aerobic exercise G2: No intervention	Three times per week for 6 months	cf-PWV	6 months
Molmen-Hansen (2012) [27]	Norway	57 subjects (G1 = 28, G2 = 29). Mean age(SD): G1 = 53.6 years (6.5), G2 = 51.3 years (9.2)	Hypertensive patients	G1: Moderate intensity continuous training G2: Standard care	Three times per week for 12 weeks	LVEF, CO	12 weeks
Nualnim (2012) [58]	USA	43 subjects (G1 = 24, G2 = 19). Mean age(SD): G1 = 58 years (9.8), G2 = 61 years (8.7)	Hypertension	G1: Swimming training G2: Attention control	Three to four days a week for 12 weeks	ASP, APP, AIx, CO, cf-PWV	12 weeks
Oliveira (2015) [59]	Portugal	86 subjects (G1 = 44, G2 = 42). Mean age: G1 = 55 years, G2 = 58.5 years	Myocardial infarction	G1: Exercise-based cardiac rehabilitation program G2: No intervention	Three sessions a week for 8 weeks	ASP, ADP, AIx, cf-PWV	8 weeks
Parnell (2002) [60]	Australia	21 subjects (G1 = 11, G2 = 10). Mean age(SD): G1 = 57 years (15), G2 = 53 years (11)	Congestive heart failure	G1: Aerobic exercise training G2: Usual lifestyle	Once a day on 5–7 days per week for 8 weeks	ASP, ADP, AIx, cf-PWV	8 weeks
Seals (2001) [61]	USA	35 subjects (G1 = 18, G2 = 17). Mean age(SD): G1 = 62 years (9), G2 = 65 years (10)	Elevated systolic pressure	G1: Walking G2: Sodium restriction	Five days a week for the 3 months	ASP, ADP, AIx, cf-PWV	3 months
Su (2011) [63]	Taiwan, China	29 subjects (G1 = 17, G2 = 12). Mean age(SD): G1 = 52 years (9), G2 = 52 years (8)	Chronic myocardial infarction	G1: Training program G2: No intervention	3 months	LVEF	3 months
Tang (2013) [64]	Canada	50 subjects (G1 = 25, G2 = 25). Mean age(SD): G1 = 65.9 years (6.4), G2 = 66.9 years (7.8)	Stroke	G1: High-intensity aerobic exercise G2: Non-aerobic Balance/Flexibility	Three times a week for 6 months	LVEF	6 months
Westhoff (2008) [65]	Germany	24 subjects (G1 = 12, G2 = 12). Mean age(SD): G1 = 66.1 years (4), G2 = 68.4 years (9.7)	Hypertension	G1: Upper-limb cycling aerobic exercise training G2: No intervention	Three times a week for 12 weeks	AIx	12 weeks
**Resistance exercise**							
Adamopoulos (2014) [34]	Belgium	33 subjects (G1 = 21, G2 = 22). Mean age(SD): G1 = 57.8 years (11.7), G2 = 58.3 years (13.2)	Chronic heart failure	G1: Muscle training and aerobic exercise G2: Aerobic exercise	Three times a week for 12 weeks	LVEF	12 weeks
Beck (2013) [37]	USA	30 subjects (G1 = 15, G2 = 15). Mean age (SD): G1 = 21.1 years (2.3), G2 = 21.6 years (3.1)	Prehypertensive subjects	G1: Resistance exercise G2: No intervention	Three days per week for 8 weeks	ASP, ADP, AIx, cf-PWV	8 weeks
Bilinska (2010) [40]	Poland	120 subjects (G1 = 60, G2 = 60). Mean age(SD): G1 = 54.1 years (5.8), G2 = 53.9 years (5)	Patients after coronary artery bypass grafting	G1: Static training G2: No intervention	Three times a week for 6 weeks	CO	6 weeks
Blumenthal (2000) [41]	USA	78 subjects (G1 = 54, G2 = 24). Mean age(SD): G1 = 46.6 years (1.2), G2 = 47.2 years (1.8)	Mild hypertension	G1: Strength and flexibility training G2: No intervention	Three supervised exercise sessions every week for 16 weeks	CO	16 weeks
Figueroa (2013) [47]	USA	25 subjects (G1 = 13, G2 = 12). Mean age(SD): G1 = 56.4 years (2.53), G2 = 55.5 years (3.46)	Hypertension	G1: Whole-body vibration exercise training G2: No intervention	Three supervised training sessions per week for 12 weeks	cf-PWV	12 weeks
Heffernan (2013) [11]	USA	21 subjects (G1 = 11, G2 = 10). Mean age(SD): G1 = 60 years (2), G2 = 63 years (3)	Hypertension	G1: Resistance exercise training G2: Inactive control group	Three days a week for 12 weeks	ASP, ADP, AIx	12 weeks
**Combined aerobic and resistance exercise**							
Acanfora (2016) [32]	Italy	40 subjects (G1 = 20, G2 = 20). Mean age(SD): G1 = 71 years (4), G2 = 71 years (3)	Chronic heart failure	G1: Exercise training G2: No intervention	Six times a week for 4 weeks	LVEF	4 weeks
Acanfora (2016) [32]	Italy	32 subjects (G1 = 16, G2 = 16). Mean age(SD): G1 = 54 years (4), G2 = 53 years (3)	Chronic heart failure	G1: Exercise training G2: No intervention	Six times a week for 4 weeks	LVEF	4 weeks
Chrysohoou (2015) [26]	Greece	72 subjects (G1 = 33, G2 = 39). Mean age(SD): G1 = 63 years (9), G2 = 56 years (11)	Chronic heart failure	G1: Combined aerobic exercise and strength exercise G2: No intervention	Three days a week for 12 consecutive weeks	LVEF, AIx, cf-PWV	12 weeks
Ehlken (2016) [45]	Switzerland	87 subjects (G1 = 46, G2 = 41). Mean age(SD): G1 = 55 years (15), G2 = 57 years (15)	Arterial hypertension	G1: Exercise training G2: No intervention	Five days a week for the following 12 weeks	CO	12 weeks
Lee (2015) [56]	South Korea	26 subjects (G1 = 14, G2 = 12). Mean age(SD): G1 = 64 years (7.4), G2 = 63 years (5.45)	Chronic post stroke hemiparesis	G1: Combined aerobic and resistance exercise G2: Standard care	Three times a week for 16 weeks	ASP, ADP, cf-PWV	16 weeks
Molmen-Hansen (2012) [27]	Norway	60 subjects (G1 = 31, G2 = 29). Mean age(SD): G1 = 52.5 years (7.4), G2 = 51.3 years (9.2)	Hypertensive patients	G1: Combined aerobic and resistance exercise G2: Standard care	Three times per week for 12 weeks	LVEF, CO	12 weeks
Stewart (2005) [62]	USA	104 subjects (G1 = 51, G2 = 53). Mean age: G1 = 63 years, G2 = 64.1 years	Hypertension	G1: Combined aerobic and resistance training G2: Usual care	Three days a week for 26 weeks	cf-PWV	6 months

**Abbreviations**: ASP, aortic systolic pressure; ADP, aortic diastolic pressure; CO, cardiac output; LVEF, left ventricle ejection fraction; AIx, augmentation index; cf-PWV, carotid-femoral pulse wave velocity.

#### Risk of bias of the selected studies

The risk of bias of the selected studies was evaluated by the PEDro scale (Table 2). The eligibility criteria, blind assessors and adequate follow-up were reported in 27 articles (71.05%), 17 articles (44.74%) and 28 articles (73.68%), respectively. Random allocation, baseline comparability, between-group comparison and point estimates and variability were reported in 38 articles (100%), 37 articles (97.37%), 36 articles (94.74%) and 36 articles (94.74%), respectively. Concealed allocation and intention to treat analysis were carried out in 4 (10.53%) and 9 articles (23.68%), respectively. In addition, there were no articles involving blinded subjects and blinded therapists.

**Table 2:** Risks of bias among the selected articles.

Article, year	Eligibility criteria	Random allocation	Concealed allocation	Baseline comparability	Blind subjects	Blind therapists	Blind assessors	Adequate follow-up	Intention to treat analysis	Between- group comparisons	Point estimates and variability
Acanfora (2016) [32]	YES	YES	NO	YES	NO	NO	NO	NO	NO	YES	YES
Acar (2015) [33]	YES	YES	NO	YES	NO	NO	NO	YES	NO	YES	YES
Adamopoulos (2014) [34]	YES	YES	NO	YES	NO	NO	NO	NO	NO	YES	YES
Aksoy (2015) [35]	YES	YES	NO	YES	NO	NO	NO	NO	NO	YES	YES
Andersen (2014) [36]	NO	YES	YES	YES	NO	NO	NO	YES	YES	YES	YES
Beck (2013) [37]	YES	YES	NO	YES	NO	NO	NO	YES	NO	YES	YES
Beer (2008) [38]	NO	YES	NO	YES	NO	NO	YES	YES	NO	YES	YES
Belardinell (1995) [39]	YES	YES	NO	YES	NO	NO	NO	YES	NO	YES	YES
Bilinska (2010) [40]	YES	YES	NO	YES	NO	NO	NO	YES	NO	YES	YES
Blumenthal (2000) [41]	YES	YES	NO	YES	NO	NO	NO	NO	YES	YES	NO
Brubaker (2009) [42]	NO	YES	NO	YES	NO	NO	YES	NO	NO	YES	YES
Chrysohoou (2015) [26]	YES	YES	NO	YES	NO	NO	NO	NO	YES	YES	YES
Donley (2014) [43]	NO	YES	NO	NO	NO	NO	NO	YES	NO	YES	YES
Dubach (1997) [44]	NO	YES	NO	YES	NO	NO	NO	YES	NO	YES	YES
Ehlken (2016) [45]	NO	YES	NO	YES	NO	NO	YES	YES	NO	YES	YES
Faulkner (2016) [46]	YES	YES	NO	YES	NO	NO	NO	YES	YES	YES	YES
Figueroa (2013) [47]	YES	YES	NO	YES	NO	NO	YES	YES	NO	YES	YES
Fu (2013) [48]	YES	YES	NO	YES	NO	NO	NO	YES	NO	YES	YES
Giallauria (2013) [49]	YES	YES	NO	YES	NO	NO	YES	YES	NO	YES	YES
Giannuzzi (2003) [50]	YES	YES	NO	YES	NO	NO	YES	YES	NO	YES	YES
Guimaraes (2010) [51]	YES	YES	NO	YES	NO	NO	YES	NO	NO	NO	YES
Hambrecht (2000) [52]	YES	YES	NO	YES	NO	NO	YES	YES	NO	YES	YES
Heffernan (2013) [11]	NO	YES	NO	YES	NO	NO	NO	NO	NO	YES	NO
Huang (2014) [53]	YES	YES	NO	YES	NO	NO	NO	YES	NO	YES	YES
Iellamo (2000) [54]	YES	YES	NO	YES	NO	NO	YES	YES	NO	YES	YES
Kitzman (2013) [25]	YES	YES	NO	YES	NO	NO	YES	YES	NO	YES	YES
Krustrup (2013) [55]	NO	YES	NO	YES	NO	NO	NO	YES	YES	YES	YES
Lee (2015) [56]	YES	YES	YES	YES	NO	NO	YES	YES	NO	YES	YES
Madden (2013) [57]	YES	YES	YES	YES	NO	NO	YES	YES	YES	YES	YES
Molmen-Hansen (2012) [27]	YES	YES	NO	YES	NO	NO	NO	NO	NO	YES	YES
Nualnim (2012) [58]	YES	YES	NO	YES	NO	NO	YES	YES	YES	YES	YES
Oliveira (2015) [59]	YES	YES	NO	YES	NO	NO	YES	YES	YES	YES	YES
Parnell (2002) [60]	YES	YES	NO	YES	NO	NO	YES	YES	NO	YES	YES
Seals (2001) [61]	NO	YES	NO	YES	NO	NO	YES	YES	NO	YES	YES
Stewart (2005) [62]	YES	YES	NO	YES	NO	NO	NO	YES	NO	YES	YES
Su (2011) [63]	NO	YES	NO	YES	NO	NO	NO	NO	NO	NO	YES
Tang (2013) [64]	NO	YES	YES	YES	NO	NO	YES	YES	YES	YES	YES
Westhoff (2008) [65]	YES	YES	NO	YES	NO	NO	NO	YES	NO	YES	YES

#### Effect of different exercise modalities on the central hemodynamics—Aortic systolic pressure (ASP)

**Fig 2:**
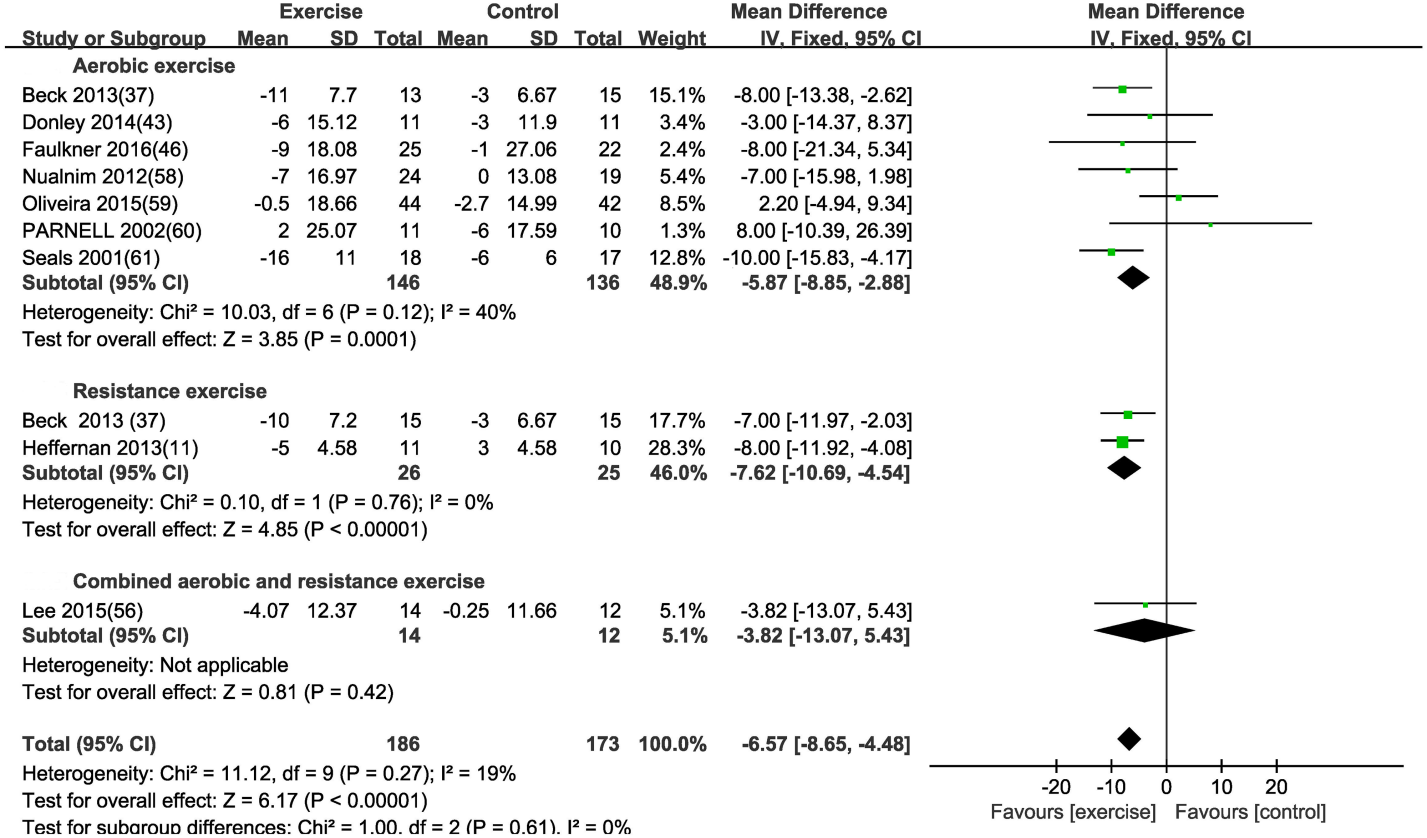
Forest plot of the change in aortic systolic pressure (ASP) in the exercise and control groups. Subgroups correspond to the exercise modalities. Squares represent the MD for each trial, and diamonds represent the pooled MD in ASP across trials. SD = standard deviation; IV = inverse variance; 95% CI = 95% confidence intervals.

Based on a fixed effects model, ASP was significantly improved by aerobic exercise [MD (95% CI) = -5.87 (-8.85, -2.88), P = 0.0001] and resistance exercise [MD (95% CI) = -7.62 (-10.69, -4.54), P<0.00001] for the exercise group when compared to the control group (Table 3 and Fig 2). There was no reduction in ASP in subjects undertaking combined exercise in patients with CVD [MD (95% CI) = -3.82 (-13.07, 5.43), P = 0.42] in this subgroup analysis due to one included study. Subgroup analyses of aerobic exercise according to age, disease, exercise duration and gender were listed in Table 4.

**Table 3:** Summary of results.

Outcome	Trials	Participant	Statistical Method	Effect Estimate	Heterogeneity	P Value
**Aerobic exercise**						
Central hemodynamics						
ASP	7 [37,43,46,58–61]	282	MD (IV, fixed, 95% CI)	-5.87 [-8.85, -2.88]	0.12	0.0001
ADP	4 [37,54, 59–60]	221	MD (IV, fixed, 95% CI)	0.06 [-2.19, 2.31]	0.21	0.96
AIx	10 [37,43,46,55,55,58–61,65]	372	MD (IV, random, 95% CI)	-2.62 [-6.51, 1.27]	<0.00001	0.19
Arterial stiffness						
Cf-PWV	8 [43,51,51,57,57,58–60]	330	MD (IV, fixed, 95% CI)	-0.42 [-0.83, -0.01]	0.95	0.04
Cardiac function						
CO	7 [41,44,48,52,53,27,58]	372	MD (IV, fixed, 95% CI)	0.36 [0.08 to 0.64]	0.43	0.01
LVEF	19 [33,35,35,36,36,38,38,39,42,44,48,48,49,50,52,25,27,63–64]	843	MD (IV, fixed, 95% CI)	3.02 [2.11, 3.93]	0.01	<0.00001
**Resistance exercise**						
Central hemodynamics						
ASP	2 [37,11]	51	MD (IV, fixed, 95% CI)	-7.62 [-10.69 to -4.54]	0.76	<0.00001
ADP	2 [37,11]	51	MD (IV, fixed, 95% CI)	-4 [-5.63, -2.37]	1	<0.00001
AIx	2 [37,11]	51	MD (IV, random, 95% CI)	0.42 [-4.55, 5.38]	0.003	0.87
Arterial stiffness						
Cf-PWV	2 [37,47]	55	MD (IV, fixed, 95% CI)	-0.26 [-0.72, 0.20]	0.18	0.27
Cardiac function						
CO	1 [41]	78	MD (IV, fixed, 95% CI)	-0.02 [-0.6 to 0.56]		0.95
LVEF	1 [34]	43	MD (IV, fixed, 95% CI)	2 [-3.26 to 7.26]		0.46
**Combined aerobic and resistance exercise**						
Central hemodynamics						
ASP	1 [56]	26	MD (IV, fixed, 95% CI)	-3.82 [-13.07 to 5.43]		0.42
ADP	1 [56]	26	MD (IV, fixed, 95% CI)	-4.2 [-11.49, 3.09]		0.26
AIx	1 [26]	72	MD (IV, random, 95% CI)	7.60 [3.75, 11.45]		0.0001
Arterial stiffness						
Cf-PWV	3 [26,56,62]	202	MD (IV, fixed, 95% CI)	-1.15 [-1.95, -0.36]	0.64	0.004
Cardiac function						
CO	1 [27]	60	MD (IV, fixed, 95% CI)	0.9 [-0.39 to 1.41]		0.0006
LVEF	3 [32,32,27]	140	MD (IV, fixed, 95% CI)	0.79 [-1.18 to 2.77]	0.54	0.43

**Abbreviations**: ASP, aortic systolic pressure; ADP, aortic diastolic pressure; cf-PWV, carotid-femoral pulse wave velocity; CO, cardiac output; LVEF, left ventricle ejection fraction.

**Table 4:** Subgroup analyses and meta-regression of effect of aerobic exercise on the central hemodynamics, arterial stiffness and cardiac function according to age, disease, exercise duration and gender.

Variable	Trials (No.)	WMD(95%CI)	p and p value^a^
**ASP**			
**Age**			
< = 50 years	3(71)	-6.10 [-10.80, -1.40]	0.01
>50 years	4(211)	-5.71 [-9.57, -1.85]	0.004
**Disease**			
hypertension	2(71)	-7.74 [-12.35, -3.12]	0.001
heart disease	4(164)	-4.20 [-8.29, -0.11]	0.04
stroke	1(47)	-8.00 [-21.34, 5.34]	0.24
**Exercise duration**			
8 weeks	4(171)	-6.03 [-9.34, -2.72]	0.0004
12 weeks	3(111)	-5.15 [-12.06, 1.75]	0.14
**Gender**			
Women	1(35)	-10.00 [-15.83, -4.17]	0.0008
trial with men and women	6(247)	-4.40 [-7.87, -0.93]	0.01
**ADP**			
**Age**			
< = 50 years	2(49)	-2.83 [-6.89, 1.22]	0.17
>50 years	2(165)	1.42 [-1.37, 4.20]	0.32
**Disease**			
hypertension	1(28)	-4.00 [-9.19, 1.19]	0.13
heart disease	3(186)	1.04 [-1.52, 3.60]	0.43
**Exercise duration**			
4 weeks	1(86)	3.30 [-1.15, 7.75]	0.15
8 weeks	2(107)	-1.15 [-4.10, 1.79]	0.44
12 weeks	1(21)	-1.00 [-7.50, 5.50]	0.76
**Gender**			
Men	1(86)	3.30 [-1.15, 7.75]	0.15
trial with men and women	3(128)	-1.13 [-3.81, 1.55]	0.41
**CO**			
**Age**			
< = 50 years	1(78)	-0.02 [-0.60, 0.56]	0.95
>50 years	6(294)	0.48 [0.16, 0.80]	0.003
**Disease**			
hypertension	3(178)	0.29 [-0.10, 0.68]	0.14
heart failure	4(194)	0.44 [0.04, 0.84]	0.03
**Exercise duration**			
8 weeks	3(91)	0.20 [-0.40, 0.80]	0.51
12 weeks	3(130)	0.59 [0.10, 1.08]	0.02
24 weeks	2(151)	0.28 [-0.14, 0.70]	0.19
**Gender**			
Men	2(98)	0.51 [-0.02, 1.04]	0.06
trial with men and women	5(274)	0.30 [-0.03, 0.64]	0.07
**cfPWV**			
**Age**			
< = 50 years	4(97)	-0.46 [-1.23, 0.31]	0.24
>50 years	4(233)	-0.40 [-0.89, 0.08]	0.1
**Disease**			
hypertension	5(201)	-0.43 [-0.93, 0.08]	0.1
heart disease	3(129)	-0.40 [-1.10, 0.30]	0.26
**Exercise duration**			
8 weeks	2(108)	-0.49 [-1.22, 0.24]	0.19
12 weeks	3(116)	-0.30 [-0.88, 0.28]	0.31
> = 16weeks	3(106)	-0.61 [-1.54, 0.32]	0.2
**Gender**			
Men	2(98)	0.51 [-0.02, 1.04]	0.06
trial with men and women	5(274)	0.30 [-0.03, 0.64]	0.07
**AIx**			
**Age**			0.981^a^
40–50 years	3(88)	-8.13 [-14.31, -1.95]	0.01
50–60 years	4(172)	-2.57 [-7.18, 2.03]	0.27
>60 years	3(106)	1.10 [-7.20, 9.40]	0.8
**Disease**			0.206^a^
hypertension	6(196)	-3.60 [-9.04, 1.85]	0.2
heart disease	4(151)	-0.13 [-3.17, 2.90]	0.93
Stroke	1(47)	-28.00 [-58.95, 2.95]	0.08
**Exercise duration**			0.133^a^
8 weeks	5(179)	-0.03 [-1.94, 1.88]	0.97
12 weeks	5(182)	-4.60 [-13.28, 4.07]	0.3
24 weeks	1(33)	-12.00 [-19.92, -4.08]	0.003
**Gender**			0.703^a^
Men	2(66)	-10.70 [-15.40, -6.01]	<0.00001
Women	1(35)	6.00 [3.35, 8.65]	<0.00001
trial with men and women	6(271)	-1.11 [-3.78, 1.56]	0.42
**LVEF**			
**Age**			0.000^a^
< = 50 years	2(76)	7.45 [4.44, 10.47]	<0.00001
>50 years	17(767)	2.58 [1.62, 3.53]	<0.00001
**Disease**			0.954^a^
hypertension	3(133)	7.47 [5.10, 9.84]	<0.00001
heart failure	9(458)	2.25 [1.10, 3.41]	0.0001
other heart disease	6(202)	4.78 [2.01, 7.55]	0.0007
Stroke	1(50)	0.00 [-2.62, 2.62]	1
**Exercise duration**			0.918^a^
<12 weeks	4(135)	1.16 [-1.45, 3.78]	0.38
12–16 weeks	9(287)	3.40 [1.89, 4.91]	<0.0001
> = 24 weeks	6(321)	3.19 [1.93, 4.46]	<0.00001
**Gender**			0.276^a^
Men	5(203)	4.91 [2.69, 7.13]	<0.0001
trial with men and women	14(640)	2.64 [1.65, 3.64]	<0.00001

^**a**^p value for the meta-regression analysis between subgroups

p value for subgroup analysis between two group

ASP, aortic systolic pressure; ADP, aortic diastolic pressure; cf-PWV, carotid-femoral pulse wave velocity; CO, cardiac output; LVEF, left ventricle ejection fraction, WMD, weighted mean difference.

#### Aortic diastolic pressure (ADP)

**Fig 3:**
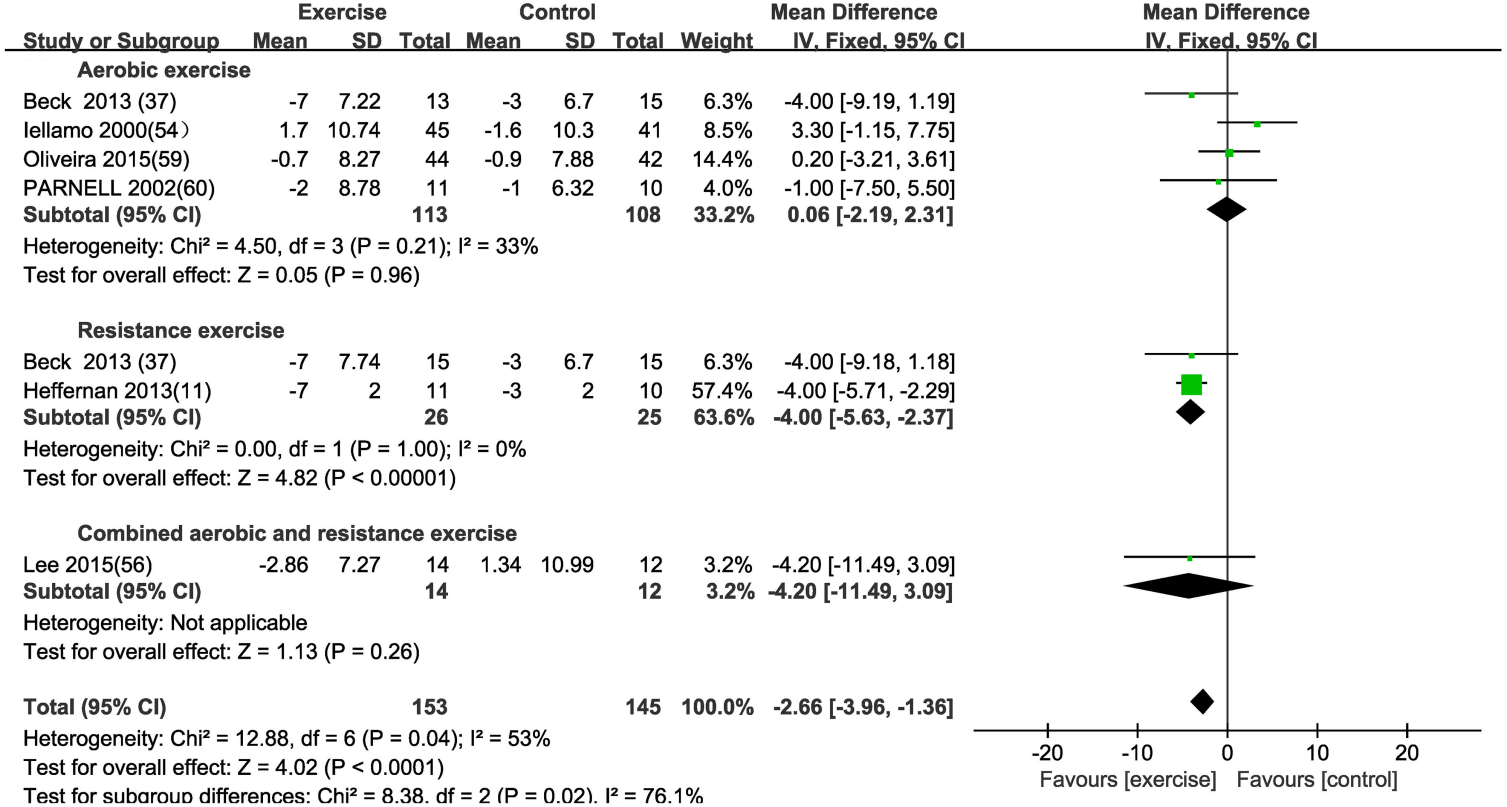
Forest plot of the change in aortic diastolic pressure (ADP) in the exercise and control groups. Subgroups correspond to the exercise modalities. Squares represent the MD for each trial, and diamonds represent the pooled MD in ADP across trials. SD = standard deviation; IV = inverse variance; 95% CI = 95% confidence intervals.

Using a fixed effects model, ADP was not significantly improved by aerobic exercise [MD (95% CI) = 0.06 (-2.19, 2.31), P = 0.96] or combined exercise [MD (95% CI) = -4.2 (-11.49, 3.09), P = 0.26] (Table 3 and Fig 3). However, Resistance exercise was found to reduce ADP by 4 mmHg [MD (95% CI) = -4 (-5.63, -2.37), P<0.001] for the exercise group when compared to the control group (Table 3 and Fig 3). Subgroup analyses of aerobic exercise according to age, disease, exercise duration and gender were reported in Table 4.

#### Augmentation index (AIx)

**Fig 4:**
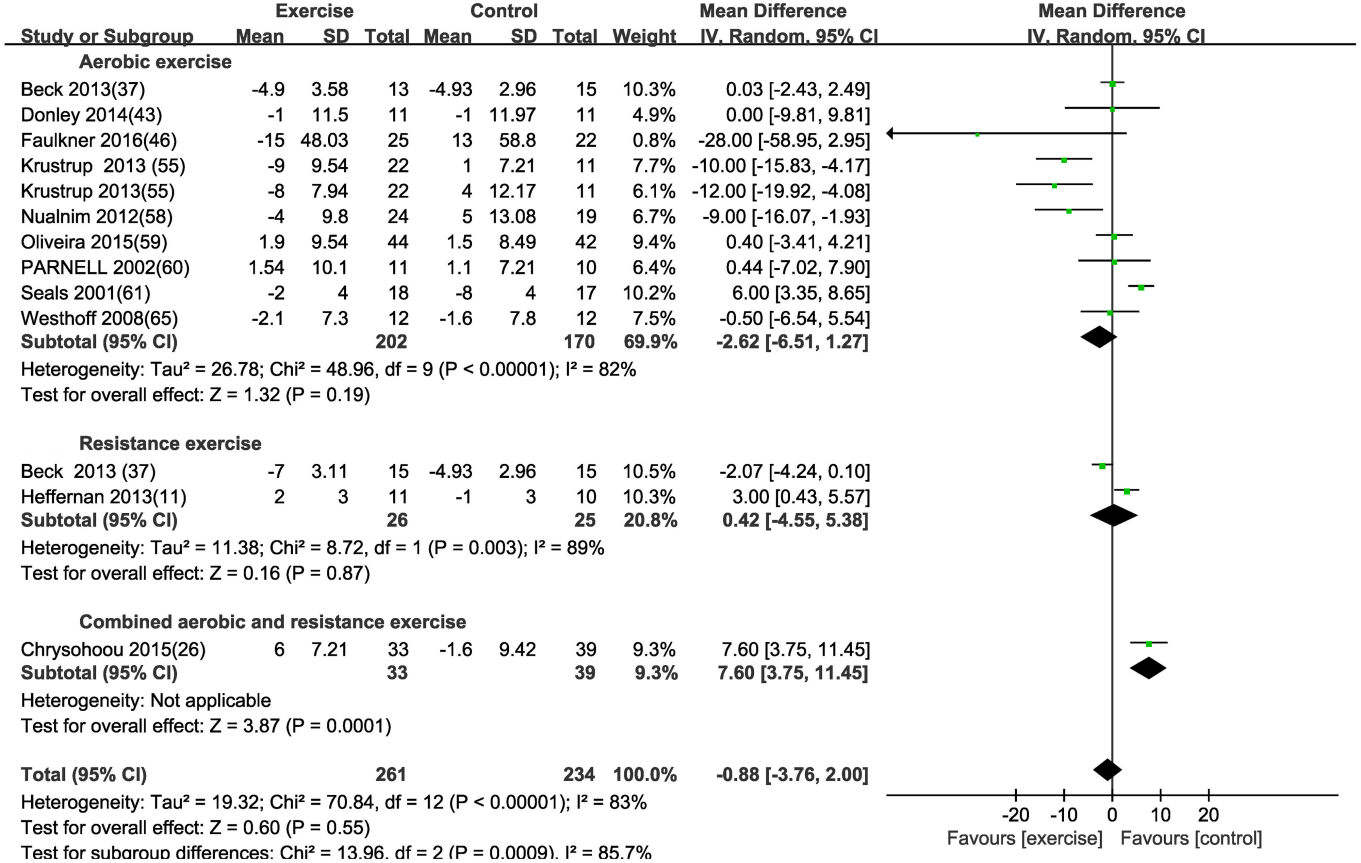
Forest plot of the change in central augmentation index (AIx) in the exercise and control groups. Subgroups correspond to the exercise modalities. Squares represent the MD for each trial, and diamonds represent the pooled MD in AIx across trials. SD = standard deviation; IV = inverse variance; 95% CI = 95% confidence intervals.

A random effects model was selected as there were larger fluctuation of the variance between each included study in AIx. There was no significant difference in AIx after aerobic and resistance exercise in CVD, while there was a significant difference in AIx after combined exercise in this subgroup analysis due to one included study (Fig 4 and Table 3). Subgroup and regression analyses of effect of aerobic exercise on AIx were conducted according to age, disease, exercise duration and gender (Table 4). AIx was significantly decreased by 24-week aerobic exercise [MD (95% CI) = -12.00 (-19.92, -4.08), P = 0.003] or in patients aged 50–60 years [MD (95% CI) = -8.13 (-14.31, -1.95), P = 0.01]. AIx was significantly increased with combined exercise in patients with chronic heart failure (CHF) when compared to a control group with one included study. According to the meta-regression analysis, AIx was not affected by age (P = 0.981), gender (P = 0.703), disease (P = 0.206) and exercise duration (P = 0.133). In this meta-analysis, of 10 trials (aerobic exercise) that measured AIx, only 4 studies (152) reported heart rate-adjusted AIx. This result found that there was no significant difference [MD (95% CI) = 2.95 (-0.77, 6.67), P = 0.12] in heart rate-adjusted AIx after aerobic exercise (S1 Fig).

#### Effect of different exercise modalities on central arterial stiffness—Carotid-femoral pulse wave velocity (cf-PWV)

**Fig 5:**
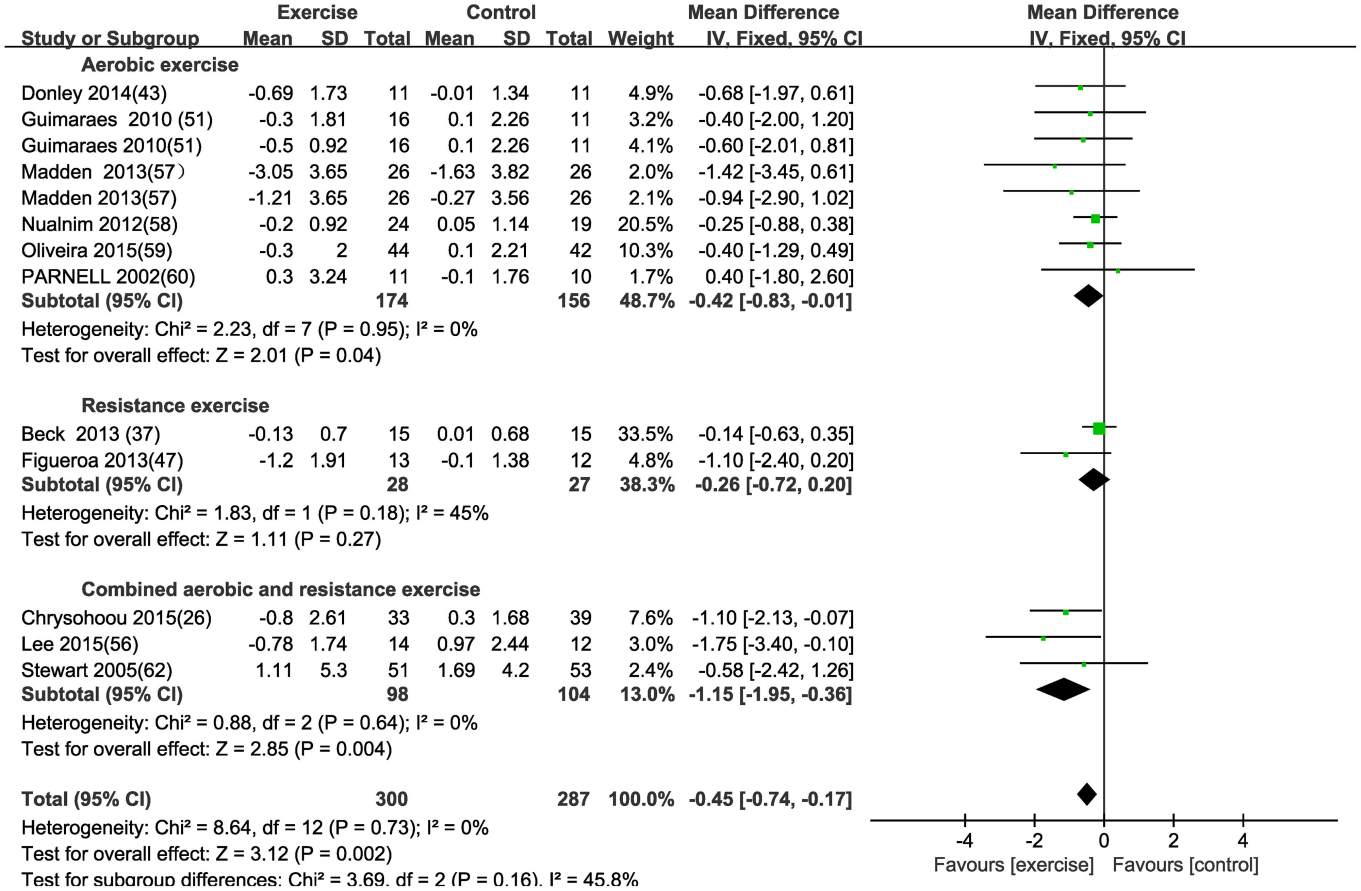
Forest plot of the change in carotid-femoral pulse wave velocity (cf-PWV) in the exercise and control groups. Subgroups correspond to the exercise modalities. Squares represent the MD for each trial, and diamonds represent the pooled MD in cf-PWV across trials. SD = standard deviation; IV = inverse variance; 95% CI = 95% confidence intervals.

There was a significant change in cf-PWV in response to aerobic exercise [MD (95% CI) = -0.42 (-0.83, -0.01), P = 0.04] and combined exercise [MD (95% CI) = -1.15 (-1.95, -0.36), P = 0.004] in patients with CVD, using a fixed effects model. However, no significant difference was found after resistance exercise [(MD (95% CI) = -0.26 (-0.72, 0.20), P = 0.27] for the exercise group compared with the control group (Table 3 and Fig 5). The result (mean difference or 95%CI or test for overall effect) was affected by one study [26] for cf-PWV after combined exercise in this sensitivity analysis. Therefore, this meta-analysis may provide weak evidence of the effect of combined exercise on cf-PWV. Subgroup analyses of aerobic exercise according to age, disease, exercise duration and gender were listed in Table 4.

#### Effect of different exercise modalities on cardiac function—Cardiac output (CO)

**Fig 6:**
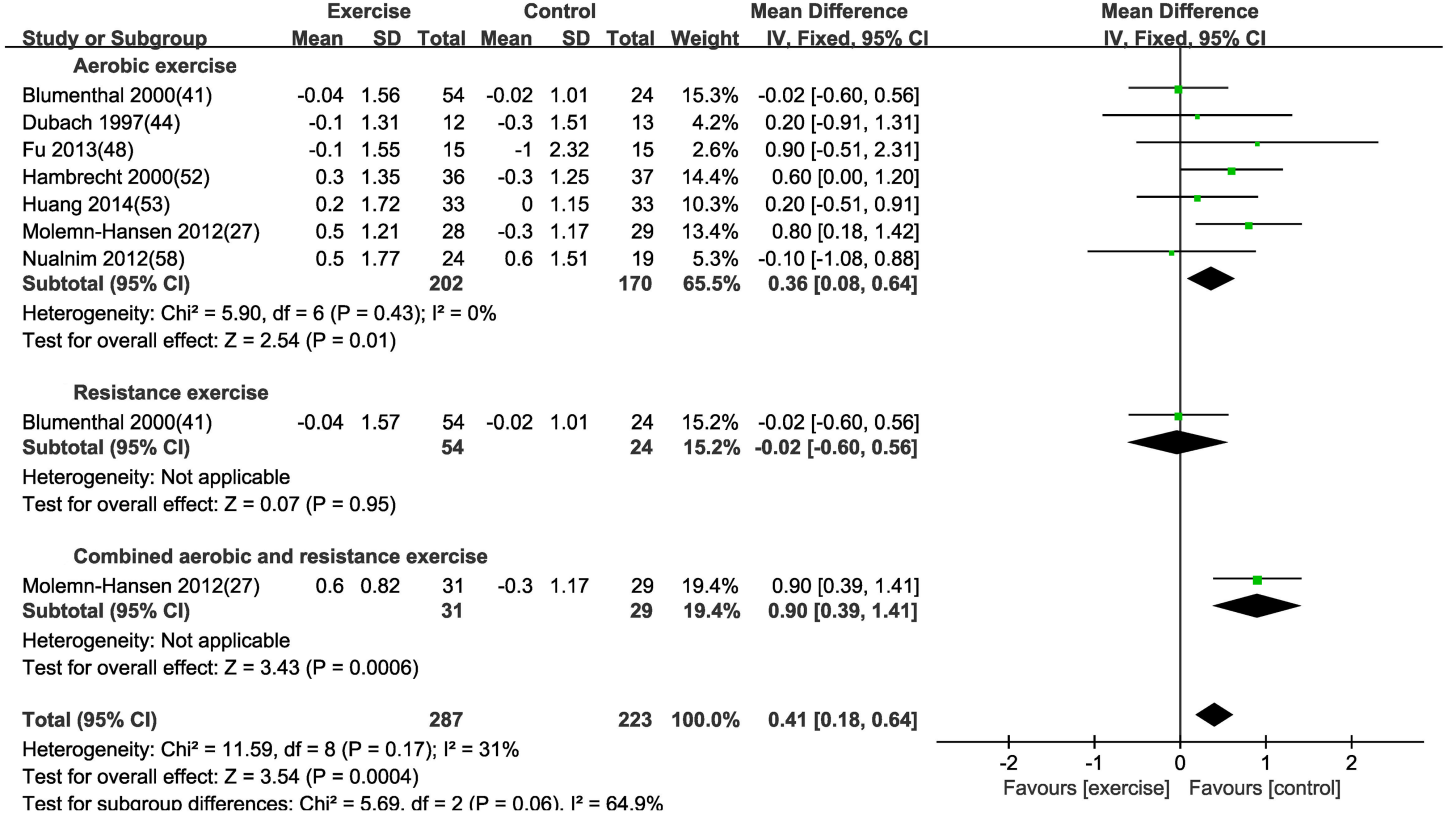
Forest plot of the change in cardiac output (CO) in the exercise and control groups. Subgroups correspond to the exercise modalities. Squares represent the MD for each trial, and diamonds represent the pooled MD in CO across trials. SD = standard deviation; IV = inverse variance; 95% CI = 95% confidence intervals.

Using a fixed effects model, seven studies with 372 patients were included to evaluate the effect of exercise on CO. Aerobic exercise and combined exercise were found to increase CO by 0.36 L/min [MD (95% CI) = 0.36 (0.08, 0.64), P = 0.01] and 0.9 L/min [MD (95% CI) = 0.9 (0.39, 1.41), P = 0.0006] in the exercise group when compared to the control group (Table 3 and Fig 6), respectively. However, CO was not significantly improved by resistance exercise in patients with CVD [MD (95% CI) = -0.02 (-0.6, 0.56), P = 0.95]. Subgroup analyses of aerobic exercise according to age, disease, exercise duration and gender were reported in Table 4. Age and disease affected the change of CO after aerobic exercise.

#### Left ventricular ejection fraction (LVEF)

**Fig 7:**
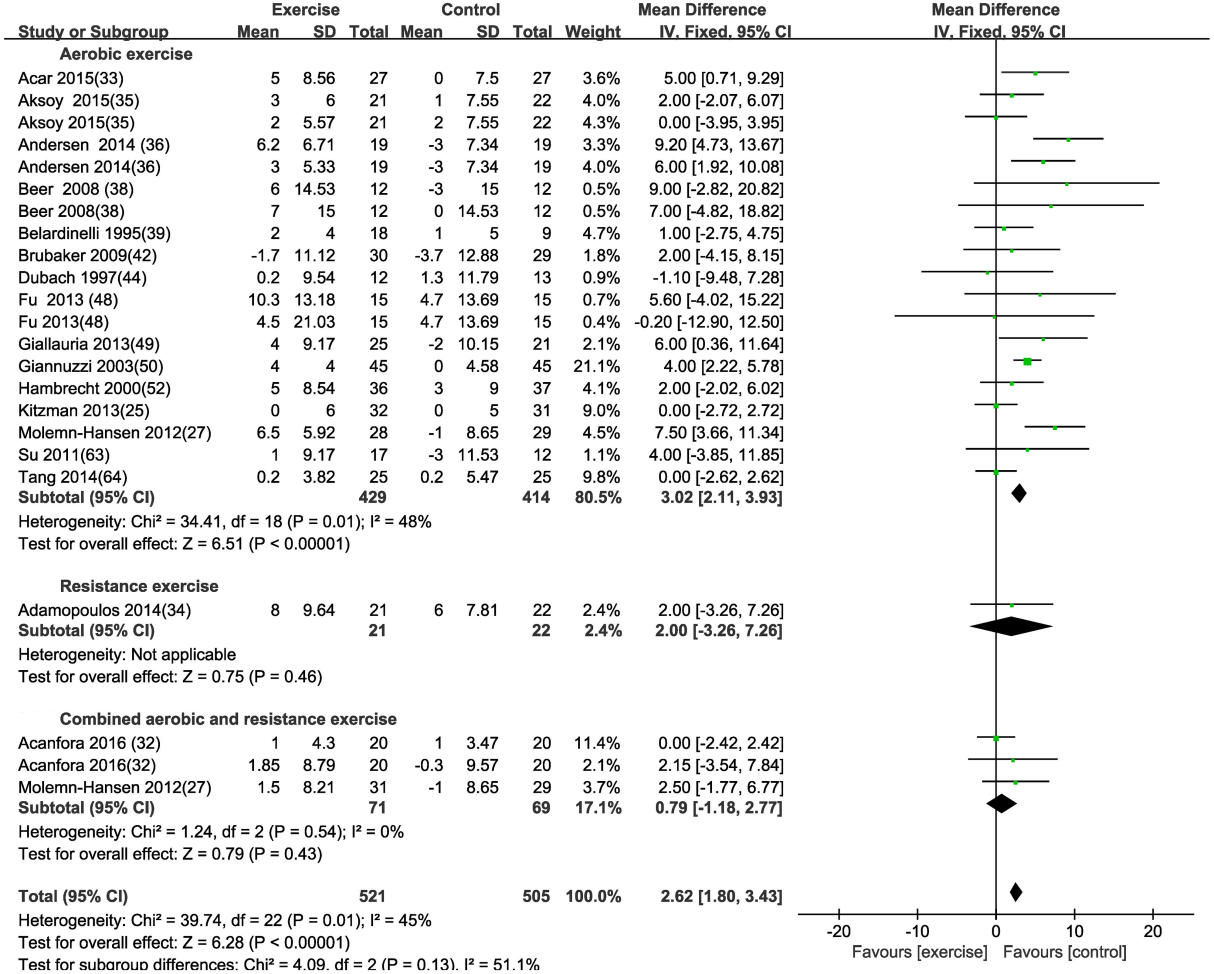
Forest plot of the change in left ventricular ejection fraction (LVEF) in the exercise and control groups. Subgroups correspond to the exercise modalities. Squares represent the MD for each trial, and diamonds represent the pooled MD in LVEF across trials. SD = standard deviation; IV = inverse variance; 95% CI = 95% confidence intervals.

**Fig 8:**
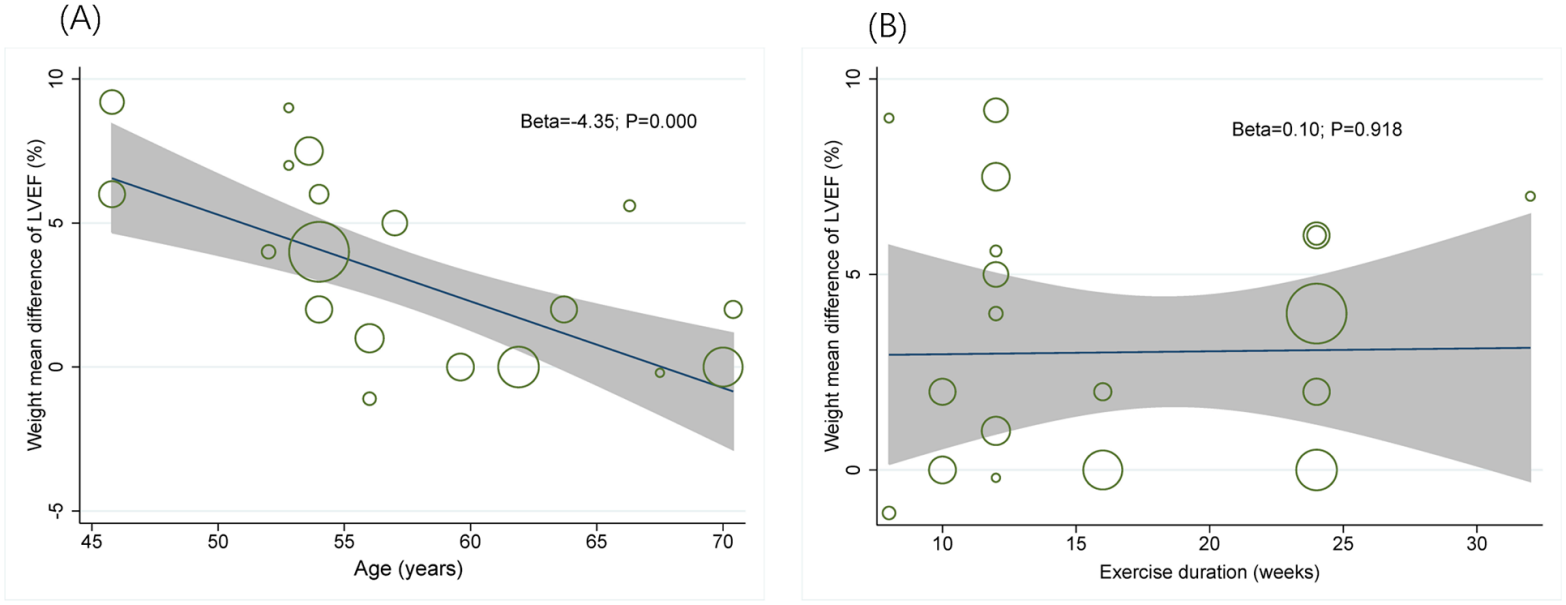
Meta-regression for exploring heterogeneity in LVEF. (A) The association between mean difference of left ventricular ejection fraction (LVEF) and different age groups with CVD. (B) The association between mean difference of left ventricular ejection fraction (LVEF) and aerobic exercise intervention of different exercise durations. (The shaded areas represent the range of confidence interval; size of the circles indicates sample size).

This meta-analysis of nineteen studies with 843 patients, using a fixed effects model, showed that aerobic exercise increased LVEF [MD (95% CI) = 3.02 [2.11, 3.93], P<0.00001], when compared to non-exercising controls (Table 3 and Fig 7). No significant difference was found after resistance exercise [MD (95% CI) = 2 (-3.26, 7.26), P = 0.46] or combined exercise [MD (95% CI) = 0.79 (-1.18, 2.77), P = 0.43] (Table 3 and Fig 7). A sensitivity analysis was conducted for LVEF after aerobic exercise, and the significance of the difference between the exercise and control groups was not changed when studies were removed 1 by 1. Meta-regression was used to explore the higher heterogeneity (I^2^ = 48%) and showed evidence of a relationship between mean difference of LVEF and age, for patients with CVD (Beta = -3.47, P = 0.000, Fig 8A and Table 4), while mean difference of LVEF was not related to changes of exercise duration (Beta = 0.59, P = 0.389, Fig 8B and Table 4), disease (Beta = -0.55, P = 0.954, Table 4) and gender (Beta = 1.96, P = 0.276, Table 4).

### Publication bias

There was no publication bias for ASP (asymmetry test P = 0.068), ADP (asymmetry test P = 0.352), CO (asymmetry test P = 0.189), AIx (asymmetry test P = 0.561) LVEF (asymmetry test P = 0.102) and cf-PWV (asymmetry test P = 0.07) according to the results of Egger’s regression test.

## Discussion

This meta-analysis, which gathered 2089 patients with CVD from 38 articles, provides evidence of the effects of exercise and differences between three types of exercise on central hemodynamics, central arterial stiffness and cardiac function. The results suggest that in patients with CVD, aerobic exercise significantly improved ASP, cf-PWV, CO and LVEF. Resistance exercise significantly reduced ASP and ADP. Combined exercise significantly improved cf-PWV and CO in patients with CVD.

We have not found any meta-analysis or systematic review that had evaluated the effects of different exercise modalities on central hemodynamics, arterial stiffness and cardiac function of patients with CVD, and therefore there was no systematic description of evidence for these differences. Previous studies have focused primarily on either aerobic or resistance exercise rather than combined, or have considered only a few measures of arterial stiffness but not those associated with central blood pressure, AIx and cardiac function. In this study, only RCTs of patients with CVD were included, and the impact of these two types of exercise, both singly and combined, on a wider range of cardiovascular variables were investigated. Therefore, this study provides a broader evaluation of the effects of different exercise modalities on central hemodynamics, central arterial stiffness and cardiac function in patients with CVD as well as their application for the rehabilitation of CVD.

In this meta-analysis, we observed significant changes, in response to aerobic and resistance exercise, in central BP, (this being more predictive for target organ damage), cardiovascular morbidity and mortality in comparison with brachial BP [66, 67] in patients with CVD. However, there was no significant difference in ASP or ADP after combined exercise in this subgroup analysis due to one included study [56]. Previous studies have supported the notion that aerobic or resistance exercise reduces ASP due to improvements in vasoactive substances and endothelial function [17, 18, 37, 68], and agreed that there was no significant difference in ADP with aerobic exercise [59]. However, a study showed unfavorable effect of resistance exercise on central blood pressure and arterial compliance [69]. The high blood pressure in response to resistance exercise may be mainly related to arterial stiffness [70]. At the same time, increased arterial stiffness was associated with the promotion of vascular smooth muscle cell growth and inflammatory cytokines, increasing the central blood pressure [71]. However, several other mechanisms for the favorable effect of resistance exercise on central blood pressure have been proposed. Croymans et al. suggested that the beneficial effect of resistance exercise on central blood pressure is due to improved endothelial function and microvascular perfusion [17]. Whereas, Heffernan et al. found that resistance exercise significantly improved central blood pressure because of its impact on the reservoir pressure, which is proportional to the volume of blood stored in the aorta, and which in turn depends on the interactions of systemic arterial compliance and impedance to outflow [11, 72]. However, Figueroa and Taaffe reported that it may be attributed to improved peripheral muscular artery dilation and peripheral vascular resistance (PVR) [18, 68]. The reductions of PVR and arteriolar tone with resistance exercise may change terminal impedance enabling greater runoff into peripheral microvascular beds during diastole (inflow< outflow) resulting in sustained reductions in reservoir pressure [72]. In the subgroup analysis of different exercise modalities, only one included study investigated effect of combined exercise on central blood pressure, with a weak evidence for this effect. Additionally, although there was no significant difference in effect of combined exercise on ASP due to one limited study (P = 0.42), different exercise modalities significantly decreased ASP according to the overall effect of total subgroup (P<0.00001).

In this study, subgroup analysis and meta-regression analysis of studies (effect of aerobic exercise on AIx) were conducted according to age, disease, exercise duration and gender. AIx was significantly decreased in response to 24-weeks aerobic exercise or in patients aged 50–60 years. However, the change of AIx was not affected by gender, disease and HR after aerobic exercise intervention. The previous work found that different durations and different age were associated with the effect of exercise training on the cardiovascular health [73]. In addition, some studies also reported that AIx may be influenced by age or disease, as arterial reservoir pressure increases rapidly with age and disease [74, 75]. Furthermore, AIx may be affected by other factors including LV afterload, exercise intensity, frequency and duration, central wave reflection, forward wave genesis and impedance [11, 13, 75, 76]. AIx was not only affected by wave reflection from pulse wave, but also affected by compliant properties of elastic arteries [77]. Combined exercise significantly increased AIx in patients with CHF in only one included study [26]. We found that aerobic exercise with resistance exercise just improved the aerobic capacity of patients with CHF [78], while a study found that increased AIx was related to improved ventricular-aortic coupling in response to combined exercise, and increased AIx and PWV can reveal the improved arterial function [26]. In this report, the result may be influenced by the fact that only one study [26] was included. Therefore, future experimental studies should investigate the AIx of patients with CHF in response to combined exercise.

This meta-analysis also reveals that aerobic and combined exercise significantly improved cf-PWV in patients with CVD. Our findings agree with those of some studies that aerobic exercise decreased cf-PWV in CVD patients or in all adults [13–15], and found that improved cf-PWV with aerobic exercise may be related to increased nitric oxide (NO) availability, higher conduit artery elastin content, decreased concentration of vasoconstrictor agents, increased oxygen uptake (VO_2)_ peak and reduction in ASP [13, 43]. However, our findings did not agree with those of previous studies which reported no significant effect of aerobic training on PWV in patients with acute myocardial infarction (MI) due to the limited exercise duration [10, 59]. In addition, some studies have reported that cf-PWV was not improved in hypertensive subjects or in patients with chronic kidney disease in response to aerobic exercise because of the high level of blood pressure regulated by sympathetic nervous system [10, 79]. Combined exercise also significantly improved cf-PWV in patients with CVD in this study. Our findings support those of Li et al. [80] that combined exercise may have favorable effect on arterial stiffness when aerobic and resistance exercise take place in the same exercise session. However, this contrasts with previous meta-analyses which found no significant difference in PWV with combined exercise [13, 14]. These meta-analyses pooled the results from subjects with disparate conditions (some were healthy, others were obese and others were CVD patients). Furthermore, peripheral and central arterial stiffness data were pooled. Montero et al. [10, 14] included non-randomized controlled trials in their analysis. We emphasize that, in this study the response of central hemodynamic variables to different exercise modalities were comprehensively evaluated, and we restricted our analysis to RCTs of patients with CVD. The different results from these meta-analyses may be related to small number of studies in subgroup analysis. In addition, Montero et al. found that cf-PWV did not decrease with combined exercise due to the limitation of resistance exercise component [14]. It was controversial and complex for effects of resistance exercise on cf-PWV [16, 17], and the effects of resistance were not only associated with exercise intensity and healthy status, but also with the changes of arterial compliance and central blood pressure regulated by sympathetic nervous system activity, and protein synthesis regulating muscle mass [14, 16, 80, 81].

Aerobic exercise also significantly improved LVEF, and both aerobic exercise and combined exercise significantly improved CO in patients with CVD. However, there was no significant difference in LVEF in response to resistance exercise. A previous meta-analysis has shown that aerobic exercise improved EF, and reversed LV remodeling in patients with HF, while the benefit was not evident with combined exercise and resistance exercise [82]. In this study, meta-regression showed that the effects of aerobic exercise on LVEF were associated with age. LVEF is significantly increased with aerobic exercise due to its impact on the release of vasoconstrictive neurohormones, hemodynamic loading, peak exercise stroke volume (SV), myocardial contractility and diastolic filling [12, 82–84]. The increased SV with exercise was associated with a reduction in TPR driven by sympathetic activity and vagal tone [85]. At the same time, myocardial contractility contributed to the increased SV in response to exercise training [84]. It was controversial whether resistance training had any beneficial effect on LVEF. Some studies found that it may be associated with increased systolic and diastolic pressure loading, while others found that it may increase LV wall stress with resistance exercise leading to decreased LV wall stress and contractile and preload reserve [12]. This study supported the findings of the previous ones that CO was significantly increased with aerobic exercise because of improved VO_2_ peak related to oxygen delivery and decreased TPR, HR and SV contributing to cardiac performance, and ventricular filling [48, 79, 86].

Although this meta-analysis has reported some novel findings concerning the disparate effects of different modalities on central hemodynamics, arterial stiffness and cardiac function in CVD, we note several limitations. First, some studies had small sample sizes, and according to the subgroup analysis of exercise modalities, only one study investigated the effects of resistance exercise on LVEF or combined exercise on ASP, ADP and AIx in this subgroup analysis. These reports had no heterogeneity results for these outcomes in the numerical results. Larger-scale, good quality RCTs are needed for further investigating the effect of exercise on CVD. Second, a total of 10 articles (26.32%) had a long-term exercise intervention period (over 6 months), therefore, the long-term effects of different types of exercise on patients with CVD were not performed in this meta-analysis. Further studies are needed to explore the pathophysiological mechanisms of the relationship among central hemodynamics, arterial stiffness and cardiac function in CVD in response to different exercise training on the basis of controlling of external variables, such as age or disease.

## Conclusions

Different exercise modalities have different effects on central hemodynamics, arterial stiffness and cardiac function in patients with CVD. Aerobic or resistance exercise significantly decreased ASP. Meanwhile, long-term aerobic exercise reduced AIx in patients with CVD. Aerobic exercise and combined exercise can effectively improve central arterial stiffness and cardiac function. The decreased central blood pressure in response to resistance exercise was mainly due to reservoir pressure reduction or improved microvascular perfusion. In contrast, improvement of central arterial stiffness and cardiac function in response to aerobic exercise and combined exercise was mainly due to their effects on cardiopulmonary fitness (VO_2_ peak related to oxygen delivery or SV) and endothelial function. However, some heterogeneity in the results of the papers considered in this review remains unexplained, partly due to a paucity of high-quality studies, especially ones concerned with the effects of combined exercise on central hemodynamics and arterial stiffness for the rehabilitation of CVD. Finally, we note that these findings have important implications in the rehabilitation of these patients, not only for the patients themselves but also for medical professionals, allowing individual treatments to be tailored to specific cardiovascular pathologies.

## Supporting information

S1 Text PRISMA 2009 checklist. (DOC)

S2 Text Search strategy. (DOCX)

S1 Fig Effect of aerobic exercise on heart rate-adjusted AIx in patients with CVD. (TIF)
