# Supplementary material for: Effects of exercise modalities on central hemodynamics, arterial stiffness and cardiac function in cardiovascular disease: Systematic review and meta-analysis of randomized controlled trials
Source: PLoS One. 2018 Jul 23;13(7):e0200829. doi: 10.1371/journal.pone.0200829 (PMC6056055; doi:10.1371/journal.pone.0200829)
Supplement: S2 Text — (DOCX) [file pone.0200829.s002.docx]

**Supplementary appendix e-1: Search strategies for all databases**

1. Search Strategy for MEDLINE (Pubmed):

#1 "Single-Blind Method"[Mesh] OR "Double-Blind Method"[Mesh] OR "Randomized Controlled Trials as Topic"[Mesh] OR "Randomized Controlled Trial" [Publication Type] OR "Intention to Treat Analysis"[Mesh] OR "Controlled Clinical Trials as Topic"[Mesh] OR "Clinical Trials as Topic"[Mesh] OR "Clinical Trial" [Publication Type] OR randomized controlled trial[Publication Type]

#2 "random*"[Text Word] OR allocation[Text Word] OR "random allocation"[Text Word] OR placebo[Text Word] OR single blind[Text Word] OR double blind[Text Word] OR "randomized controlled trial*"[Text Word] OR RCT[Text Word]

#3 #1 OR #2

#4 animals NOT humans

#5 #3 NOT #4

#6 chronic disease[Mesh] OR “chronic disease” [Mesh] OR "cardiovascular diseases"[Mesh] OR "heart disease"[Mesh] OR stroke[Mesh] OR "non-communicable diseases" OR "non-infectious diseases" OR "non-transmissible diseases" OR "heart"[Title/Abstract] or stroke[Title/Abstract] OR Atherosclerosis[Title/Abstract] OR vascular disease[Title/Abstract]

#7 cardiac function [Mesh] OR arterial stiffness [Title/Abstract] OR “central hemodynamic*” [Title/Abstract] OR aortic function [Title/Abstract] OR central blood pressure [Title/Abstract] OR central systolic pressure [Title/Abstract] OR central pulse pressure [Title/Abstract] OR wave reflection [Title/Abstract] OR myocardial perfusion [Title/Abstract] OR cardiac output [Title/Abstract] OR pulse wave velocity [Title/Abstract]

#8 #5 AND #6 AND #7

#9 resistance exercise [Mesh] OR resistance exercise [Title/Abstract] OR resistance training [Title/Abstract] OR strength training [Title/Abstract]

#10 aerobic exercise [Title/Abstract] OR endurance exercise [Title/Abstract] OR dynamic exercise[Text Word] OR aerobic training[Title/Abstract] OR exercise training[Text Word] OR physical activity[Title/Abstract] OR habitual exercise[Text Word] OR sports [Mesh] OR swimming [Text Word] OR football [Text Word/ Abstract] OR dancing[Text Word] OR yoga[Text Word] OR taichi [Text Word]

#11 Combine aerobic exercise and resistance exercise [Title/Abstract] OR exercise [Title/Abstract] OR combine resistance and endurance exercise [Title/Abstract] OR combine aerobic and resistance exercise [Title/Abstract] OR resistance, aerobic and combination training [Title/Abstract] OR comprehensive exercise program [Title/Abstract]

#12 #9 OR #10 OR #11

#13 #8 AND #12

2. Search Strategy for Cochrane Library

#1 "random*" or allocation or "random allocation" or placebo or single blind or double blind or "randomized controlled trial*" or RCT or "clinical trial*"

#2 randomized controlled trial:pt or clinical trial:pt

#3 Aerobic exercise:ti OR swimming:ti OR football:ti OR dancing:ti OR yoga:ti OR endurance exercise:ti OR dynamic exercise:ti OR aerobic training:ti OR exercise training:ti OR physical activity:ti OR habitual exercise:ti OR resistance exercise:ti OR resistance training:ti OR strength training:ti OR Combine aerobic exercise and resistance exercise:ti OR exercise:ti OR combine resistance and endurance exercise:ti OR combine aerobic and resistance exercise:ti OR resistance, aerobic and combination training:ti OR group training:ti OR comprehensive exercise program:ti

#4 chronic disease:ti,ab,kw OR cardiovascular diseases:ti,ab,kw OR stroke:ti,ab,kw OR heart disease:ti,ab,kw OR non-communicable diseases:ti,ab,kw OR non-infectious diseases:ti,ab,kw OR non-transmissible diseases:ti,ab,kw OR Atherosclerosis:ti,ab,kw OR vascular disease:ti,ab,kw

#5 cardiac function:ti,ab,kw OR arterial stiffness:ti,ab,kw OR central hemodynamic*:ti,ab,kw OR aortic function:ti,ab,kw OR central blood pressure:ti,ab,kw OR central systolic pressure:ti,ab,kw

OR wave reflection:ti,ab,kw OR myocardial perfusion:ti,ab,kw OR cardiac output:ti,ab,kw OR pulse wave velocity :ti,ab,kw

#6 #1 and #2 and #3 and #4 and #5

3. Search Strategy for web of science

#1 TS=("random*" OR allocation OR "random allocation" OR placebo OR single blind OR single blind method OR double blind OR double blind method OR "randomized controlled trial*" OR "randomised controlled trial*" OR "RCT" OR "clinical trial*")

#2 TI=(aerobic exercise OR aerobic training OR exercise training OR physical activity OR endurance exercise OR dynamic exercise OR habitual exercise OR exercise OR swimming OR football OR dancing OR yoga OR basketball)

#3 TI=( resistance exercise OR resistance training OR strength training)

#4 TI=( Combine aerobic exercise and resistance exercise OR exercise OR combine resistance and endurance exercise OR combine aerobic and resistance exercise OR resistance, aerobic and combination training OR group training OR comprehensive exercise program)

#5 #2 OR #3 OR #4

#6 TS=( chronic disease OR "cardiovascular diseases" OR "heart disease" OR stroke OR "non-communicable diseases" OR "non-infectious diseases" OR " atherosclerosis " OR " vascular disease ")

#7 TS=( cardiac function OR " arterial stiffness " OR " central hemodynamic*" OR aortic function OR " central blood pressure" OR " central systolic pressure"OR " wave reflection" OR " pulse wave velocity")

#8 #1 AND #5 AND #6 AND #7

Timespan=All years. Databases=SCI-EXPANDED, SSCI, A&HCI, CPCI-S, CPCI-SSH.

4. Search Strategy for CINAHL (Ebsco)

S1 MH("Random Assignment" OR "Placebos" OR "Placebo Effect" OR "Single-Blind Studies" OR "Double-Blind Studies" OR "Triple-Blind Studies" OR "Randomized Controlled Trials" OR "comparative studies" OR "Evaluation Research" OR "Prospective Studies" OR "crossover Design" OR "Prospective Studies" OR "Clinical Trials" OR "Clinical Trial Registry")

S2 TX(random$ OR allocation OR "random allocation" OR placebo$ OR single blind OR double blind OR "randomi?ed controlled trial*" OR "controlled clinical trial*" OR "comparative study" OR "evaluation stud*" OR "follow-up stud*" OR "prospective stud*" OR "cross-over stud*" OR control$ OR prospectiv$ OR volunteer$ OR "RCT" OR "clinical trial*")

S3 PT( randomized controlled trial OR "clinical trial*")

S4 S1 OR S2 OR S3

S5 AB(aerobic exercise OR aerobic training OR exercise training OR physical activity OR endurance exercise OR dynamic exercise OR habitual exercise OR swimming OR football OR dancing OR yoga)

S6 AB( resistance exercise OR resistance training OR strength training)

S7 AB( Combine aerobic exercise and resistance exercise OR exercise OR combine resistance and endurance exercise OR combine aerobic and resistance exercise OR resistance, aerobic and combination training OR group training OR comprehensive exercise program)

S8 S5 OR S6 OR S7

S9 AB( chronic disease OR "cardiovascular diseases" OR "heart disease" OR stroke OR "non-communicable diseases" OR "non-infectious diseases" OR " atherosclerosis " OR " vascular disease ")

S10 AB( cardiac function OR " arterial stiffness " OR " central hemodynamic*" OR aortic function OR " central blood pressure" OR " central pulse pressure" OR " wave reflection " OR "cardiac output" OR "pulse wave velocity" )

S11 S8 AND S9 AND S10

5. Search Strategy for Sciencedirect:

#1 "Single-Blind Method" OR "Double-Blind Method" OR "Randomized Controlled Trials as Topic" OR "Randomized Controlled Trial" OR "Intention to Treat Analysis" OR "Controlled Clinical Trials as Topic" OR "Clinical Trials as Topic" OR "Clinical Trial" OR randomized controlled trial

#2 “chronic disease” OR “cardiovascular disease” OR “stroke” OR "non-communicable diseases" OR "non-infectious diseases" OR “heart disease” OR “vascular disease” OR " atherosclerosis"

#3 “central hemodynamic*” OR “arterial stiffness” OR “central blood pressure” OR “pulse pressure” OR “wave reflection” OR “pulse wave velocity” OR “cardiac output”

#4 “aerobic exercise” OR “aerobic training” OR “endurance exercise” OR “dynamic exercise” OR “training exercise” OR “habitual exercise” OR “resistance exercise” “resistance exercise” OR “strength exercise” OR “combined aerobic and resistance exercise” OR “combined aerobic exercise and resistance exercise” OR “exercise program” OR “physical activity”

#5 #1 AND #2 AND #3 AND #4
