# Supplementary figures and images for: Effects of exercise modalities on central hemodynamics, arterial stiffness and cardiac function in cardiovascular disease: Systematic review and meta-analysis of randomized controlled trials
Source: PLoS One. 2018 Jul 23;13(7):e0200829. doi: 10.1371/journal.pone.0200829 (PMC6056055; doi:10.1371/journal.pone.0200829)

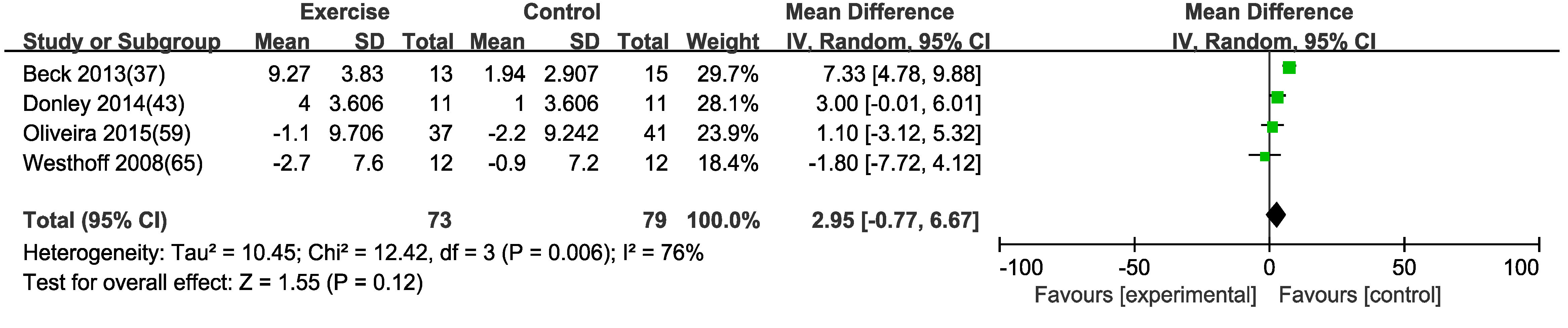

Supplement: S1 Fig — (TIF) [file pone.0200829.s003.tif]
